# Increased Thrombin-Activatable Fibrinolysis Inhibitor and Decreased Tissue Factor Pathway Inhibitor and Thrombomodulin Levels in Children with Hypothyroidism

**DOI:** 10.4274/Jcrpe.652

**Published:** 2012-09-11

**Authors:** Bülent Alioğlu, Nevin Kılıç, Enver Şimşek, Yıldız Dallar

**Affiliations:** 1 The Ministry of Health of Turkey, Ankara Training and Research Hospital, Department of Pediatric Hematology, Director of the Hematology Laboratories, Ankara, Turkey; 2 The Ministry of Health of Turkey, Ankara Training and Research Hospital, Department of Pediatrics, Ankara, Turkey; 3 The Ministry of Health of Turkey, Ankara Training and Research Hospital, Department of Pediatric Endocrinology, Ankara, Turkey

**Keywords:** hypothyroidism, thrombin-activatable fibrinolysis inhibitor, tissue factor pathway inhibitor, thrombomodulin

## Abstract

**Objective:** We determined the profile of coagulation/fibrinolytic and vascular endothelial cell function parameters including plasminogen activator inhibitor (PAI) and thrombin-activatable fibrinolysis inhibitor (TAFI), tissue factor pathway inhibitor (TFPI), thrombomodulin (TM), and tissue plasminogen activator (tPA) levels in children with hypothyroidism.

**Methods:** Forty children with hypothyroidism aged 0-16 months who presented for the first time to our hospital and 29 age-and sex-matched healthy controls were enrolled in the study. All coagulation tests were performed with ELISA method. One year after Na-L-thyroxine treatment, the study parameters were re-evaluated in 25 euthyroid children out of the 40 patients diagnosed with hypothyroidism.

**Results:** Although no significant effect was detected regarding PAI antigen (Ag) and tPA Ag, the levels of TAFI, TM, and TFPI were consistent with subclinical hypercoagulability and hypofibrinolysis. There was a significant increase in TAFI Ag levels and a significant decrease in TFPI Ag and TM Ag levels in hypothyroid patients compared to healthy controls. As a result of correlation tests, the largest impact of hypothyroidism on coagulation system was on TFPI. In accordance with these findings, TAFI Ag levels decreased and TFPI Ag and TM Ag levels increased with hormonal replacement therapy.

**Conclusions:** Increased TAFI and decreased TFPI and TM in patients with hypothyroidism may indicate a potential hypercoagulable and hypofibrinolytic state as well as possible endothelial dysfunction, which may increase the risk of atherosclerotic and atherothrombotic complications. Thyroid hormone levels should also be checked in patients with a predisposition to coagulation, and thyroid replacement therapy should be initiated.

**Conflict of interest:**None declared.

## INTRODUCTION

Various abnormalities of coagulation and fibrinolytic system have been reported in patients with thyroid dysfunction (1,2,3,4,5,6). These abnormalities range from subclinical laboratory findings to clinically significant coagulopathies and, more rarely, major hemorrhagic fatal thromboembolic events (3,4,5,6). Although it has been generally agreed that hypothyroid patients have a bleeding tendency (7,8), the more recent literature findings have evidenced that the interaction between thyroid dysfunction and hemostasis is more complex than initially believed (9). 

Increased plasminogen activator inhibitor (PAI) and thrombin-activatable fibrinolysis inhibitor (TAFI) levels and decreased tissue factor pathway inhibitor (TFPI), thrombomodulin (TM), and tissue plasminogen activator (tPA) levels have been associated with several thrombotic conditions like venous thromboembolism, ischemic stroke, and thrombotic thrombocytopenic purpura (10,11,12,13,14,15).

Although several studies have reported that coagulation and fibrinolytic system is disturbed in patients with hypothyroidism, the levels of plasma PAI antigen (Ag), TAFI Ag, TFPI Ag, TM Ag, and tPA Ag have been less investigated in patients with hypothyroidism (16,17). Therefore, in a case-control study, we aimed to investigate the profile of coagulation/fibrinolytic and vascular endothelial cell function parameters including PAI, TAFI, TM, TFPI, and tPA in children with hypothyroidism.

## METHODS

**Selection of the Study Groups**

Forty children with hypothyroidism aged 0-16 months who presented for the first time to our Pediatric Endocrinology Department were enrolled in the study. The control group consisted of age- and sex-matched healthy children who presented to the pediatric outpatient clinic for growth and development follow-up or counseling.

Data recorded included the chronological age, anthropometric features [height (cm), weight (kg), body mass index (BMI)], height standard deviation score (SDS), weight SDS, BMI SDS, presence of concomitant diseases and medication history of both patient and control groups. BMI was calculated as weight (kg)/height (m)2. SDSs of height, weight and BMI were calculated separately according to national references. None of the patients had any concomitant disease except for hypothyroidism. Neither the patients nor the controls were on drugs affecting the lipid metabolism and/or homeostasis. At diagnosis, risk factors for coagulation and thromboembolism were excluded in the patient group. 

The diagnosis of hypothyroidism was established based on clinical and hormonal data. Cases with a low free thyroxine (fT4) levels according to the age references and high levels of thyroid-stimulating hormone (TSH) (> 10 mU/L) were recognized as having primary hypothyroidism, whereas patients whose fT4 level was lower than normal references for the age group and those with low, normal or slightly above normal levels of TSH were recognized as central hypothyroidism. Hence, 27 of the patients had primary and 13 had central hypothyroidism. Twenty-five of the patients with primary hypothyroidism were diagnosed as congenital hypothyroidism and 2 with iodine deficiency-induced hypothyroidism.

One year after Na-LT4 treatment, the study parameters were re-evaluated in 25 euthyroid children out of the 40 patients diagnosed with hypothyroidism. 

This study was approved by the Training Planning and Coordination Board (protocol number 0176).

**Laboratory Methods**

All tests were conducted in the Hematology Laboratories of the Ministry of Health of Turkey, Ankara Training and Research Hospital. For all tests, venous blood samples were collected from both patient and control groups in the morning between 08^00^ and 09^00^ hour into vacutainer tubes (Vacutainer®, Beckton Dickinson, USA). Two mL of blood was taken into vacutainer tubes without anticoagulant for thyroid function tests (TSH and fT4) and 4 mL of blood was taken into standard tubes with 0.5 mL (1 volume) of 0109 M trisodium citrate for coagulation tests. Platelet-poor plasma was obtained by centrifugation at 3500 g at 10 °C for 20 minutes. The plasma was stored at -80 °C until analysis.

Following daily internal quality control and calibration tests, all laboratory tests were performed using original test kits of each device. Beckman Coulter Unicell® DXI 800 (Beckman Coulter Inc, USA) was used for the analysis of thyroid hormones. Age-specific reference ranges were defined for thyroid hormone tests (18). 

All coagulation tests including PAI Ag, TAFI Ag, TFPI Ag, TM Ag, and tPA Ag were performed with ELISA using commercial kits of American Diagnostica. According to the manufacturer’s instruction, the normal ranges are 4-43 ng/mL for PAI Ag, 0.2-2 μg/mL for TAFI Ag, 75-120 ng/mL for TFPI Ag, 2.73-5.35 ng/mL for TM Ag, and 3.9-4.7 ng/mL for tPA Ag.

**Statistical Analysis**

Statistical analysis of the data was performed using SPSS for Windows 11.5 software package. The distribution of continuous variables was tested for normality with the Shapiro-Wilk test. The descriptive statistics were presented as mean±SD or median (interquartile range) for continuous variables and as number of cases and (%) for categorical variables. 

The significance of the mean difference between the hypothyroid group and the control group was assessed using the student’s t-test, whereas the significance of the median difference between the hypothyroid and control groups was assessed by the Mann-Whitney U test. Pearson’s chi-square test was used to analyze categorical variables. Spearman’s correlation test was used to determine the relationship between continuous variables.

The dependent t-test or the Wilcoxon signed-rank test was used to assess the presence or absence of a statistically significant change between baseline and final measurements of the patients in the case group. Multivariate linear regression analysis was used to identify the clinical variables most associated with the changes of thyroid hormones. Variables with p<0.25 in the univariate analyses were entered in the multivariate regression model and were considered candidate variables. 

Multivariate linear regression analysis was performed to determine whether the most significant clinical variables identified by the gradual regression analysis maintained their significant effects on the changes of thyroid hormones after adjustment for diagnosis, age, sex, and BMI SDS values. The regression coefficient and 95% confidence interval were calculated for each variable. As thyroid hormones did not show normal distribution, logarithmically converted data were used in the regression analyses. A p-value of <0.05 was considered statistically significant.

## RESULTS

**Demographic Features**

Table 1 summarizes the clinical features of children with hypothyroidism and healthy controls. There were no differences between the groups regarding demographic characteristics such as age, sex, height, weight, BMI, height SDS, weight SDS and BMI SDS.

**Thyroid Function Tests**

The comparison of basal thyroid function tests between the patient and control groups showed both significantly higher TSH levels and lower fT4 values in the primary hypothyroid children group (p<0.001) (Table 2). When comparing the pre- and post-treatment changes of thyroid hormones in hypothyroid patients, a statistically significant decrease was observed in TSH levels, whereas fT4 levels were found to be significantly increased (p<0.001) in hypothyroidism. In central hypothyroidism, thyroid hormone levels also normalized. 

**Coagulation Tests**

No significant differences were found between the hypothyroid children and the controls in terms of PAI Ag and tPA Ag. No significant differences were found between pre- and post-treatment levels of either PAI Ag or tPA Ag (Table 3). 

However, statistically significant increase was found in TAFI Ag in hypothyroid children when compared to controls. TAFI Ag levels were significantly decreased after hormone replacement. TFPI Ag and TM Ag levels were found significantly different between hypothyroid children and controls. In addition, significant increase was observed regarding TFPI Ag and TM Ag levels after treatment. 

Spearman’s correlation test was used to determine the relationship between thyroid function tests and the study variables (Table 4). According to this test, there was a negative correlation between TFPI Ag and TSH. However, TFPI Ag was positively correlated with total T4 and fT4 levels. 

## DISCUSSION

The influence of hypothyroidism on hemostasis has been studied but is still not well understood. Contradictory results regarding hemostasis disorders have been obtained in previous studies. Various hemostatic disorders, ranging from subclinical laboratory abnormalities to clinically important hemostasis disorders have also been reported (4,19). 

The influence of hypothyroidism on hemostasis is controversial; both hypocoagulable and hypercoagulable states have been reported (2). Increased levels of fibrinogen, fibrinopeptide A, antithrombin, TFPI, and factors (F) VII, FVIII, FIX, FX, FXII, FXIII, von Willebrand factor (vWF) Ag, vWF ristocetin co-factor (vWF: RCo), and decreased fibrinolytic activity (increased PAI, alpha 2-antiplasmin and decreased D-dimer levels) in moderate hypothyroidism and increased fibrinolytic activity (lower tPA, PAI and alpha 2-antiplasmin levels) in severe hypothyroidism have been shown in previous studies (2,4,5,7,9,16,20,21,22,23).

Up to date, most of the studies investigating the effect of hypothyroidism on coagulation factors such as TAFI, TM, PAI, tPA, and TFPI were conducted in adult patients (2,4,5,6,7,9,16,20,21,22,23). Limited data have been reported about the effect of childhood hypothyroidism on coagulation system. To the best of our knowledge, this is the first study investigating the effect of childhood hypothyroidism on TAFI, TM, PAI, tPA, and TFPI.

This study showed that untreated children with hypothyroidism are prone to subclinical hypercoagulability. Although no significant effect was detected regarding PAI Ag and tPA Ag, the levels of TAFI, TM, and TFPI were consistent with subclinical hypercoagulability and hypofibrinolysis. In accordance with these findings, significant increase was found in TAFI Ag levels and significant decrease was found in TFPI Ag and TM Ag in hypothyroid children compared to healthy controls. Our thesis was confirmed by the finding that TAFI Ag levels decreased, and TFPI Ag and TM Ag levels increased on LT4 replacement treatment. 

The results of the present study confirm previous publications on the effect of hypothyroidism on TAFI and TFPI. An adult study conducted by Erem et al (24) also showed that patients with hypothyroidism are prone to hypercoagulability and hypofibrinolysis. The authors suggested that these disturbances of the hemostatic system may contribute to the excess mortality due to cardiovascular disease seen in patients with hypothyroidism. However, another study suggested that TFPI and PAI levels were significantly affected by hyperthyroid state. The authors showed that in hypothyroid and subclinical hypothyroid patients, there were no significant differences in TFPI and tPA (16).

In severe hypothyroidism, an increase in fibrinolytic activity was also reported (a decrease in alpha2-antiplasmin, tPA, and PAI-1, and an increase in D-dimer); thus, a tendency toward bleeding was observed (16). In a previous study, Ozcan et al (16) have reported that plasma TFPI levels were higher in patients with hypothyroidism compared to patients with subclinical hypothyroidism. Gullu et al (7) have also reported that there was a decrease in platelet count, a prolongation of the bleeding time, coagulation time, PT, and aPTT, and a decrease in FVIII and vWF activities. These abnormalities improved during the euthyroid period after LT4 therapy. In a very recent study, Akinci et al (17) have demonstrated that TAFI Ag levels were markedly higher in patients with overt and subclinical hypothyroidism compared to controls. In that study, a positive correlation was determined between TAFI Ag levels and the degree of thyroid failure. An increase in TAFI Ag levels in hypothyroidism is thought to be related with either a decrease in TAFI clearance or an increase in its production in the adipose tissue and endothelium. 

In previous studies, it has been shown that the TM concentration in hypothyroidism did not differ from that in control group (25,26). However, in the present study, plasma TM levels were significantly lower in children with hypothyroidism than in normal healthy children. 

According to recent knowledge, the relationship between thyroid diseases and hemostasis is more complex than assumed. We suggest that direct and indirect effects of hypothyroidism on the synthesis and action of coagulation factors and on changes in blood viscosity may play a role in the pathogenesis of coagulopathies (19). 

In conclusion, we found some important differences in the hemostatic parameters between children with hypothyroidism and healthy controls. Increased TAFI and decreased TFPI and TM in these patients may indicate a potential hypercoagulable and hypofibrinolytic state as well as possible endothelial dysfunction, which may increase the risk of atherosclerotic and thrombotic complications. Thyroid hormone levels should also be checked in patients with a predisposition to coagulation, and thyroid replacement therapy should be initiated if necessary. Nevertheless, future large-scale studies are needed to investigate the effects of hypothyroidism on the coagulation system.

## Figures and Tables

**Table 1:**
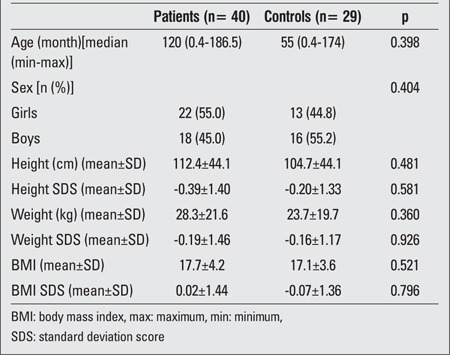
Demographic features of the children with hypothyroidism and healthy controls

**Table 2:**
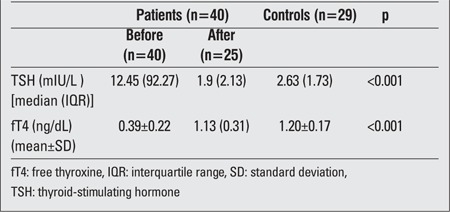
The thyroid function test results for the two study groups

**Table 3:**
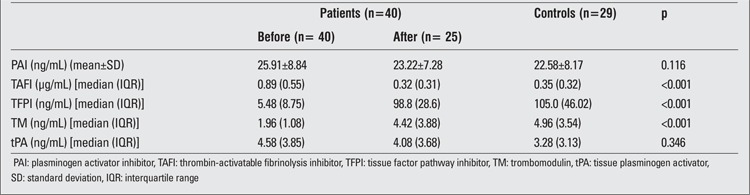
Coagulation parameter results for the hypothyroid children and healthy controls

**Table 4:**
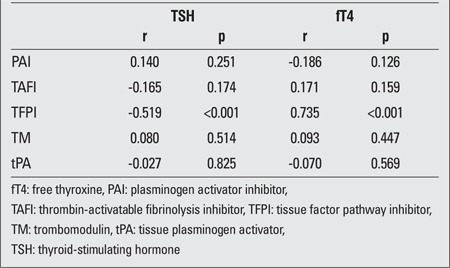
The relationship between thyroid function tests and laboratory tests: coefficients and significant values

## References

[1] Erem C, Ersoz HO, Karti SS, Ukinc K, Hacihasanoglu A, Deger O, Telatar M (2002). Blood coagulation and fibrinolysis in patients with hyperthyroidism. J Endocrinol Invest.

[2] Erem C, Kavgaci H, Ersoz HO, Hacihasanoglu A, Ukinc K, Karti SS, Deger O, Telatar M (2003). Blood coagulation and fibrinolytic activity in hypothyroidism. Int J Clin Pract.

[3] Erem C (2006). Blood coagulation, fibrinolytic activity and lipid profile in subclinical thyroid disease: subclinical hyperthyroidism increases plasma factor X activity. Clin Endocrinol (Oxf).

[4] Squizzato A, Romualdi E, Buller HR, Gerdes VE (2007). Clinical review: thyroid dysfunction and effects on coagulation and fibrinolysis:a systematic review. J Clin Endocrinol Metab.

[5] Franchini M (2004). Hemostasis and thyroid diseases revisited. J Endocrinol Invest.

[6] Marongiu F, Cauli C, Mariotti S (2004). Thyroid, hemostasis and thrombosis. J Endocrinol Invest.

[7] Gullu S, Sav H, Kamel N (2005). Effects of levothyroxine treatment on biochemical and hemostasis parameters in patients with hypothyroidism. Eur J Endocrinol.

[8] Myrup B, Bregengard C, Faber J (1995). Primary haemostasis in thyroid disease. J Intern Med.

[9] Franchini M (2006). Hemostatic changes in thyroid diseases. Hematology.

[10] Tilburg NH, Rosendaal FR, Bertina RM (2000). Thrombin activatable fibrinolysis inhibitor and the risk for deep vein thrombosis. Blood.

[11] Eichinger S, Schonauer V, Weltermann A, Minar E, Bialonczyk C, Hirschl M, Schneider B, Quehenberger P, Kyrle PA (2004). Thrombin-activatable fibrinolysis inhibitor and the risk for recurrent venous thromboembolism. Blood.

[12] Leebeek FW, Goor MP, Guimaraes AH, Brouwers GJ, Maat MP, Dippel DW, Rijken DC (2005). High functional levels of thrombin-activatable fibrinolysis inhibitor are associated with an increased risk of first ischemic stroke. J Thromb Haemost.

[13] Montaner J, Ribo M, Monasterio J, Molina CA, Alvarez-Sabin J (2003). Thrombin-activable fibrinolysis inhibitor levels in the acute phase of ischemic stroke. Stroke.

[14] Abumiya T, Yamaguchi T, Terasaki T, Kokawa T, Kario K, Kato H (1995). Decreased plasma tissue factor pathway inhibitor activity in ischemic stroke patients. Thromb Haemost.

[15] Kobayashi M, Wada H, Wakita Y, Shimura M, Nakase T, Hiyoyama T, Nagaya S, Minami N, Nakano T, Shiku H (1995). Decreased plasma tissue factor pathway inhibitor levels in patients with thrombotic thrombocytopenic purpura. Thromb Haemost.

[16] Ozcan MA, Comlekci A, Demirkan F, Yuksel F, Sari I, Demir T, Ozsan GH, Oruk G, Yesil S, Undar B (2003). Plasma levels of free tissue factor pathway inhibitor in patients with various thyroid disorders. Thromb Res.

[17] Akinci B, Comlekci A, Ali Ozcan M, Demir T, Yener S, Demirkan F, Yuksel F, Yesil S (2007). Elevated thrombin activatable ?brinolysis inhibitor (TAFI) antigen levels in overt and subclinical hypothyroid patients were reduced by levothyroxine replacement. Endocr J.

[18] Griffin JE, Griffin JE, Ojeda SR (2004). The thyroid. Textbook of endocrine physiology.

[19] Hofbauer LC, Heufelder AE (1997). Coagulation disorders in thyroid diseases. Eur J Endocrinol.

[20] Marongiu F, Biondi G, Conti M, Murtas ML, Mameli G, Sorano GG, Martino E (1992). Is a hypercoagulable state present in hypothyroidism. Thromb Haemost.

[21] Chadarevian R, Bruckert E, Ankri A, Beucler I, Giral P, Turpin G (1998). Relationship between thyroid hormones and plasma D-dimer levels. Thromb Haemost.

[22] Chadarevian R, Bruckert E, Leenhardt L, Giral P, Ankri A, Turpin G (2001). Components of the fibrinolytic system are differently altered in moderate and severe hypothyroidism. Clin Endocrinol Metab.

[23] Muller B, Tsakiris DA, Roth CB, Guglielmetti M, Staub JJ, Marbet GA (2001). Haemostatic profile in hypothyroidism as potential risk factor for vascular or thrombotic disease. Eur J Clin Invest.

[24] Erem C, Ucuncu O, Yilmaz M, Kocak M, Nuhoglu I, Ersoz HO (2009). Increased thrombin-activatable fibrinolysis inhibitor and decreased tissue pathway inhibitor in patients with hypothyroidism. Endocrine.

[25] Soma M, Maeda Y, Matsuura R, Sasaki I, Kasakura S, Saeki Y, Ikekubo K, Ishihara T, Kurahachi H, Sasaki S, Tagami T, Nakao K (1997). Study of serum thrombomodulin (TM) levels in patients with hper- or hypo-thyroidism. Rinsho Byori.

[26] Nagasaki T, Inaha M, Hiura Y, Tahara H, Kumeda Y, Shirakawa K, Onoda N, Ishikawa TT, Ishimuta E, Nishizawa Y (2005). mal thyroid function following levothroxine replacement therapy. Biomed Pharmacother.

